# Chronic Corticosterone Administration-Induced Mood Disorders in Laboratory Rodents: Features, Mechanisms, and Research Perspectives

**DOI:** 10.3390/ijms252011245

**Published:** 2024-10-19

**Authors:** Hao Wang, Xingxing Wang, Huan Wang, Shuijin Shao, Jing Zhu

**Affiliations:** 1 School of Integrative Medicine, Shanghai University of Traditional Chinese Medicine, Shanghai 201203, China; wanghao199607@163.com (H.W.); xingxingwang@shutcm.edu.cn (X.W.); huanwangshutcm@outlook.com (H.W.); shaoshuijin@163.com (S.S.); 2 Shanghai Institute of Traditional Chinese Medicine for Mental Health, Shanghai 201108, China

**Keywords:** glucocorticoid, hypothalamus–pituitary–adrenal axis, cognitive function, mineralocorticoid receptor, glucocorticoid receptor

## Abstract

Mood disorders mainly affect the patient’s daily life, lead to suffering and disability, increase the incidence rate of many medical illnesses, and even cause a trend of suicide. The glucocorticoid (GC)-mediated hypothalamus–pituitary–adrenal (HPA) negative feedback regulation plays a key role in neuropsychiatric disorders. The balance of the mineralocorticoid receptor (MR)/glucocorticoid receptor (GR) level contributes to maintaining the homeostasis of the neuroendocrine system. Consistently, a chronic excess of GC can also lead to HPA axis dysfunction, triggering anxiety, depression, memory loss, and cognitive impairment. The animal model induced by chronic corticosterone (CORT) administration has been widely adopted because of its simple replication and strong stability. This review summarizes the behavioral changes and underlying mechanisms of chronic CORT administration-induced animal models, including neuroinflammatory response, pyroptosis, oxidative stress, neuroplasticity, and apoptosis. Notably, CORT administration at different doses and cycles can destroy the balance of the MR/GR ratio to make dose-dependent effects of CORT on the central nervous system (CNS). This work aims to offer an overview of the topic and recommendations for future cognitive function research.

## 1. Introduction

The HPA axis consists of the hypothalamus, pituitary gland, and adrenal gland, and is essential in stress regulation and neuroendocrine homeostasis by regulating stress-related hormones, corticotropin-releasing hormone (CRH), adrenocorticotrophic hormone (ACTH), and glucocorticoid ((GC), cortisol in humans and CORT in rodents) [1]. Under stressful conditions, the neurons in the paraventricular nucleus (PVN) of the hypothalamus are activated, initiating the secretion of CRH, and then CRH acts on the pituitary gland, prompting the synthesis and release of ACTH, which enters into the circulation and stimulates the adrenal glands to release GC. Simultaneously, GC provides a negative feedback regulation of PVN CRH and pituitary ACTH through glucocorticoid receptors (GRs). Furthermore, the HPA axis can also be activated by some cytokines, such as interleukin-1 (IL-1), interleukin-6 (IL-6), and tumor necrosis factor-α (TNF-α). The release of noradrenaline and/or serotonin is associated with the HPA-activating activity of these cytokines [2]. Notably, the serum GC level is often used to evaluate the HPA axis function.

Mood disorders are closely associated with HPA axis dysfunction, especially severe GC fluctuation [3]. The serum GC has a strong circadian rhythm, peaking at the onset of activity (cortisol in the early morning in humans, and corticosterone in the evening in rodents) [4,5]. Elevated morning cortisol precedes depression in adolescence [6], blunted morning cortisol also may induce depression [7]. GC is essential in stress regulation, and chronic GC administration contributes to the impairment of GR-mediated HPA axis feedback invalidity and brain structural changes, causing anxiety, depression, memory loss, and cognitive impairment. GC excess exists in many neuropsychiatric disorders, including anxiety, major depressive disease, and clinical post-traumatic stress disorder [8].

GC can activate two steroid hormone receptors, MR and GR, to exert biological effects; however, MR has a 10-fold higher affinity for endogenous GC than GR. Under physiological conditions, GC generally activates MR predominantly in the limbic system. Under stressful conditions, high GC level also activates GR [9]. GR is mostly activated under high serum GC concentration, interacting with GC in the cytoplasm. Then, the GR/GC complex is translocated to the nucleus and binds to GC response elements (GREs) to regulate neuroendocrine and immune systems by modulating gene transcription [10]. FK506-binding protein 5 (FKBP5), a molecular chaperone, can influence the stress response by regulating the sensitivity of GR to affect this process and finetune the balance of MR/GR [11]. Serum- and GC-inducible kinase 1 (SGK1) can potentiate and maintain the activation of GR to regulate stress responses by increasing GR phosphorylation and GR nuclear translocation [12].

MR and GR are widely found in the central nervous system (CNS), and are especially abundant in the hippocampus (HPC), hypothalamus, and cortex, with expression on both neurons and glial cells. Both MR and GR play key roles in neuroplasticity, and MR can also evoke synaptic response [13]. They are essential for the development of CNS and cognitive function regulation. Under a traumatic brain injury, the administration of CORT replacement at a dose of 0.3 mg/kg enhanced MR expression, then promoted neuronal survival and improved spatial memory [14]. However, excess CORT administration at a dose of 40 mg/kg increased the expression of FKBP5 and phosphorylated SGK1 in the HPC, which further influenced the functions of CNS GR [15]. Simultaneously, chronic excess CORT administration at a dose of 20 mg/kg could also downregulate the expression of GR and disrupt serum GC homeostasis [16]. Thereby, chronic GC administration of different doses can disrupt the balance of the MR/GR ratio to make dose-dependent effects of GC on the CNS [17].

The balance of MR/GR plays a crucial role in maintaining the homeostasis of the neuroendocrine system. Excess CORT administration at a dose of 30 mg/kg decreases the ratio of MR/GR and increases the cell apoptosis rate, impairing spatial learning ability [17]. Like this, many patients with neuropsychiatric disorders suffer from hypercortisolemia, which further destroys the function of the HPA axis and affects cognitive functions. Existing research suggests that chronic excess GC administration induces cognitive impairment in preclinical studies. Hence, this review summarizes behavioral changes and investigates the underlying central mechanisms in chronic CORT administration-induced cognitive function animal models.

## 2. Chronic CORT Administration-Induced Cognitive Impairment

Behavioral tests are widely used to define whether a model is successfully established in neurological function research. Chronic CORT administration in rodents could cause behavioral changes, including anxiety-like behaviors, depression-like behaviors, and memory loss behaviors. Elevated plus maze (EPM), elevated zero maze (EZM), and light/dark test (LDT) are often used to evaluate anxiety-like behaviors [18], and forced swim test (FST), sucrose preference test (SPT), and tail suspension test (TST) are applied to test depression-like behaviors [19]. Furthermore, open field test (OFT), conditioned place preference (CPP), novel object recognition test (NOR), Y-maze test (YMT), Morris water maze task (MWM), and other tests are also used to evaluate cognitive and memory capabilities [20]. The changes in behaviors induced by the chronic CORT administration are summarized in Table 1. From this table, we found that long-term CORT administration mice and rats exhibited significant anxiety-like behaviors, depression-like behaviors, and memory loss.

## 3. Chronic CORT Administration Is Detrimental to the Neuroinflammatory Response and Neural Cell Pyroptosis

**Figure 1:**
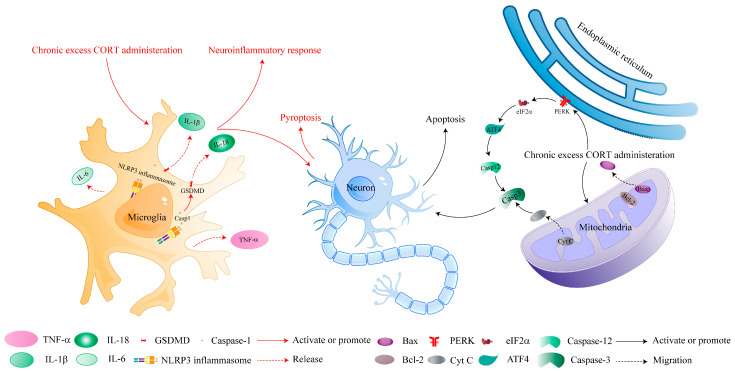
The molecular mechanism of neuroinflammatory response, pyroptosis, and apoptosis induced by chronic excess CORT administration. Chronic excess CORT administration can activate microglia to trigger a neuroinflammatory response by releasing proinflammatory cytokines. IL-18 and IL-1β can also trigger neural cell pyroptosis. In turn, chronic excess CORT administration causes neural cell apoptosis via mitochondria and ER stress pathways.

The inflammatory response is divided into the alarm phase, the mobilization phase, and the resolution phase. GC plays a significant role in these phases. Notably, GC administration was widely used to inhibit the production of inflammatory mediators and proinflammatory cytokines in the alarm phase in the clinic [108], and long-term and excess CORT administration can reverse these changes. Chronic excess CORT administration at a dose of 20 mg/kg significantly enhanced the expression of proinflammatory cytokines in the HPC and serum, including IL-1β, IL-6, and TNF-α, accompanied by remarkable depressive-like behaviors [45] (Figure 1). Simultaneously, serum interleukin-10 (IL-10), an anti-inflammatory cytokine, was downregulated [36].

NLRP3 (NLR family, pyrin domain containing 3) inflammasomes consist of NLRP3, ASC (apoptosis-associated speck-like protein containing CARD), and pro-caspase-1, which contribute to the release of IL-1β and interleukin-18 (IL-18) [109,110]. Of special interest, chronic CORT administration at a dose of 20 mg/kg could activate NLRP3 inflammasomes via increasing NLRP3, caspase-1, and ASC expression, and then enhancing microglia activation in both the HPC and medial prefrontal cortex (mPFC) [36,51] (Figure 1).

Cell pyroptosis, a caspase-1-dependent programmed cell death, mainly plays a remarkable role in neuroinflammatory response [111]. Caspases, cysteine proteases, are associated with apoptosis, maintaining neural physiological functions. Pyroptosis executor gasdermin D (GSDMD) can promote the secretion of IL-18 and IL-1β via forming a non-selective pore at the cell membrane, which needs a caspase-1-mediated cleavage of GSDMD [112,113]. Excess CORT administration at a dose of 20 mg/kg significantly increased the level of IL-1β, IL-18, and cleaved GSDMD in the HPC, which triggered neural cell pyroptosis (Figure 1).

In addition, nuclear factor-κB (NF-κB) is a transcriptional activator of the NLRP3 inflammasome. IL-1β enhances NF-κB pathway activation to induce depression-like behaviors via promoting p65 binding to the NF-κB site. The interaction of NLRP3 and NF-κB could enhance the release of inflammatory factors, leading to pyroptosis [43], and chronic CORT administration could give rise to this process.

## 4. Chronic Excess CORT-Induced Oxidative Stress Contributes to Cognitive Dysfunction

Oxidative stress (OS) in CNS is associated with cognitive impairment. Nuclear factor erythroid 2-related factor 2 (NRF2) mediated the expression of antioxidant genes, and chronic excess GC administration impaired NRF2-medicated antioxidant response by excessive GR signaling activation [114]. Furthermore, a chronic excess GC administration also induces long-lasting changes in cerebellar granule cells, further rendering OS injury in CNS [115]. Sirtuin (SIRT) 3 is a soluble protein in the mitochondrion contributing to mitochondrial functions and OS resistance [116]. SIRT3 makes glutamate dehydrogenase (GDH) and isocitrate dehydrogenase 2 (IDH2) available for tricarboxylic acid cycle regulation [117]. Of special interest, chronic CORT administration at a dose of 20 mg/kg inhibited the activities of SIRT3, GDH, and IDH2 expression. Additionally, chronic excess CORT administration also has been reported in the cytochrome oxidase (Cox)1, Cox4, NADH-ubiquinone oxidoreductase (ND)1, ND2, ND4, ND6, ATP synthase subunit beta, and ATP synthase F0 subunit 6 expression restrain [66], which indicates that CORT overdose administration might damage the mitochondrion functions by inhibiting oxidation-reduction reaction and limiting ATP synthesis in vivo. Decreased ATP production has also been involved in anxiety [118] and depression [119] formation, reportedly.

Reactive oxygen species (ROS), a derivative of oxygen molecules, is highly relevant to intracellular OS for multiple diseases. Mitochondria dysfunction restrained energy production, generated ROS, and induced stress-induced neuron apoptosis [120]. It has been reported that excess CORT administration at a dose of 40 mg/kg could also inhibit the viability of superoxide dismutase (SOD) expression, an endogenous antioxidant enzyme, which is the first line against ROS to protect our bodies [121]. ROS enhances cell damage via many pathways, including lipid peroxidation and enzyme inactivation. Malondialdehyde (MDA), a metabolite of lipid peroxidation, demonstrates the degree of lipid peroxidation damage. The level of MDA in the HPC was significantly upregulated by CORT administration at a dose of 40 mg/kg [73]. Therefore, OS damage in the CNS could be triggered by chronic excess GC administration, causing cognitive dysfunction.

## 5. Chronic Excess CORT Administration Affects Neuroplasticity

Under physiological conditions, GC/GR homeostasis contributes to neuroplasticity maintenance in the CNS; however, excess CORT administration suppresses neuronal synaptic growth. CORT injection at a dose of 10 μM triggered the number and length of dendritic spine reduction and synaptic function injury [122], which severely destructed the neurogenesis and synaptic plasticity.

Brain-derived neurotrophic factor (BDNF) plays a significant role in adult neurogenesis, neuronal maturation, and synaptic plasticity by binding to tropomyosin-related kinase B [123]. The *BDNF* gene in rodents exhibited a complex genomic structure, consisting of nine exons (I to IX) [124]. GR can bind to a specific DNA region upstream of exon IV to repress the expression of BDNF [125]. Chronic CORT administration remarkably decreased the level of BDNF in the HPC and anterior cingulate cortex, which destroyed synaptic plasticity [126]. These pathological changes manifested the relationship between GR modification and BDNF pathways. Apart from the areas of the brain, BDNF levels were also significantly decreased in the serum under excess CORT administration [127].

In the ventral and dorsal dentate gyrus (DG) area, CORT administration at a dose of 20 mg/kg caused filopodia-, thin-, and stubby-shaped spines and total spine density decrease. Simultaneously, CORT also led to hippocampal synaptic dysfunction because it inhibited the expression of postsynaptic density protein 95 (PSD-95), α-amino-3-hydroxyl-5-methyl-4-isoxazole-propionate (AMPA) receptor subunits 1 (GluA1), and synapsin in the HPC [128,129,130]. PSD-95, GluA1, and synapsin are three synaptic proteins, which are necessary for spinogenesis and synaptogenesis [60,131]. Additionally, chronic CORT administration at a dose of 20 mg/kg caused the levels of calpain 2 (Capn2), vesicle-associated membrane protein 7 (Vamp7), and C-type natriuretic peptide (Cnp) to decrease in the HPC. Capn2, Vamp7, and Cnp, three neurite growth-related genes, are involved in angiogenesis, neurogenesis, and neurite growth. Of special interest, the brain region-specific response in vivo after excess CORT administration could cause the opposite changes of these genes among different brain regions. Capn2 declined in the HPC but rose in the PFC under the CORT administration. Similarly, Vamp7 declined in the HPC but rose in the raphe nuclei [132]. This may be related to the neuron heterogeneity among different brain regions.

Microglia can update synapses at the correct time via presynaptic selective partial phagocytosis and postsynaptic spine head filopodia induction, which helps to maintain normal synaptic connections [133]. Similarly, astrocytes also play a critical role in the response to early stress. Mer tyrosine kinase (MERTK) is a member of the tyrosine kinase receptor family, which identifies and eliminates unnecessary synaptic connections [134]. Excess cortisol administration at a dose of 30 μM can activate MERTK transcription by an astrocytic GR-MERTK pathway in human cortical organoids to enhance synapse phagocytosis leading to excessive excitatory synapse elimination. On the contrary, due to the lower level of GR during the early post-natal development stage, microglia do not take part in stress hormone-induced synapse elimination [135].

GC also has a profound effect on synaptic plasticity via regulating AMPA receptor trafficking [136]. The extent of MR and GR occupation can modulate AMPA receptor subunit mRNAs [137]. GluA1 and GluA2 subunits are two AMPA receptor subunits. Notably, low-CORT administration at a dose of 5 mg/kg can increase the expression of mature BDNF, nectin3, GluA1, and GluA2 subunits in the HPC to promote cognitive function and enhance spatial learning ability [138]. Nectin3, a cell adhesion molecule, can regulate the development and plasticity of DG granule cells to improve long-term memory but not short-term memory [139]. This observation is different from the excess CORT-induced animal model. It is worthy to further exploit that the balance of the MR/GR ratio can determine the dose-dependent effects of CORT.

## 6. Chronic CORT Administration Induces Apoptosis in the CNS

Cell apoptosis often occurs via three pathways: mediated by mitochondria [140], endoplasmic reticulum (ER) stress [141], and death receptors [142]. Firstly, the outer mitochondrial membrane is permeabilized by signal stimulation, and a diverse variety of pro-apoptotic factors are released to the cytosol, such as cytochrome c. These pro-apoptotic factors in the cytosol induce caspase cascade. Notably, the outer mitochondrial membrane permeabilization (MOMP) could be modulated by proteins from the Bcl-2 family, mitochondrial lipids, and proteins that regulate bioenergetic metabolite flux [143]. Bcl-2 family members contain both pro-apoptotic and anti-apoptotic proteins. Bcl-2 is an anti-apoptotic protein in the upstream of mitochondria. Bax is a pro-apoptotic protein facilitating pore formation that induces MOMP. Both the pro-apoptotic and anti-apoptotic protein homeostasis is core in apoptosis modulation. Excess CORT administration at a dose of 30 mg/kg made GR hyperactive, raised the level of Bax, and increased the Bax/Bcl-2 ratio, which severely impaired hippocampal structure and functions. Additionally, low-CORT administration at a dose of 0.3 and 3 mg/kg restored the activation of MR and significantly decreased the level of cleaved caspase-3 and the number of apoptotic hippocampal cells [17]. This further demonstrates that the balance of the MR/GR ratio is determined by the dose-dependent effects of GC levels, and ultimately affects the CNS cell apoptosis.

Experiencing various endogenous or exogenous noxious stimuli, the misfolded proteins accumulated in the ER [144], and long-term or severe ER stress, trigger the unfolded protein response directly, leading to cell apoptosis. The protein kinase RNA-like ER kinase (PERK)/eukaryotic translation initiation factor-2α (eIF2α)/activating transcription factor 4 (ATF4) pathway plays a significantly important role in this process. PERK, a type of I trans-membrane protein, is composed of an ER luminal stress sensor and a cytosolic protein kinase domain. PERK dissociates from the ER molecular chaperone glucose-regulated protein 78, triggering trans-autophosphorylation when cells are stimulated. Activated PERK can phosphorylate downstream eTF2α at serine 51, reducing the influx of protein into the ER and increasing the selective translation of the mRNA encoding the transcription factor ATF4 [145,146]. ATF4 can encode cAMP response element-binding transcription, which activates caspase-12 and downstream caspase-3 and causes cell apoptosis [147]. It is reported that chronic CORT administration at a dose of 20 mg/kg raised the mRNA levels of caspase-12 and the protein expression of cleaved caspase-3 in the ER, leading to neural cell apoptosis and memory loss [61] (Figure 1).

## 7. Conclusions

Lots of stress-based animal models are established for the analysis of the underlying mechanisms of neuropsychiatric disorders, including the chronic mild stress model, chronic social defeat stress model, unpredictable maternal separation combined with unpredictable maternal stress model, and chronic CORT administration-induced cognitive impairment animal model. Due to its simplicity and stability, the chronic CORT administration-induced cognitive impairment animal model has been used in fundamental research [148].

MR and GR are widely distributed throughout the body, especially the stress regulation center, and play an essential role in stress regulation and cognitive functions. The HPC has rich MR and GR distribution, which is a sensitive area for stress and a high-level regulatory center for the HPA axis stress response. The HPC can inhibit the stress response of the HPA axis and promote the recovery of the hyperactive HPA axis function back to baseline levels under stressful conditions by regulating the neuron activity of PVN. GR activation could enhance the growth of pyramidal neuron dendrites in the HPC, contributing to memory consolidation and long-term memory [149]. Similarly, PFC plays a significant role in emotional generation, appraisal, and regulation. CORT administration at a dose of 35 μg/mL eliminated the expression of GR in the mPFC and affected optimal decision making [150]. The basolateral amygdala (BLA) is a recipient of negative and positive stimuli, including the basal and lateral nuclei. The basal nuclei mainly receive mPFC input, while the lateral nuclei receive sensory thalamic cortical input. The lateral nuclei also receive input from the HPC, ventral striatum, and entorhinal cortex together with the basal nuclei. GR was reported to affect the priming effect of the BLA on dentate long-term potentiation [151]. Paraventricular nucleus MR and GR expression are related to HPA axis function, which has a central role in regulating stress homeostasis. Chronic excess GC administration could enhance HPA axis dysfunction and impair cognitive functions. Central amygdala GR also affects anxiety and fear behavior regulation [152].

GC/GR/MR expression in the CNS is essential in cognitive function regulation, making chronic GC administration animals available for elucidating the underlying mechanisms and finding potential targets in cognitive dysfunction research. But there are still some unresolved issues. Firstly, the peripheral MR/GR’s effects on the CNS have not been elucidated. The adrenal gland is the main producer of stress hormones, and chronic GC administration affects adrenal function. Whether the adrenal function affects cognitive functions under chronic excess GC intervention remains unclear. Consistently, GC could affect thymic T-cell differentiation in the earliest events [153], and the effect of thymic T-cell function on cognitive functions has not been observed under stressful conditions.

Additionally, MR/GR is expressed both in the neuron and glia. Although the GC effect on neuronal and glial functions has been recognized, the GC effect on circuit modulation and the crosstalk of neurons and glial cells under excess GC levels has not been fully interpreted. Cell heterogeneity in the CNS affects the GC effect on cognitive function modulation. It is necessary to fully understand the roles of glia–neuron crosstalk in chronic excess GC administration animal models to fully explain the underlying mechanisms of neuropsychiatric diseases.

Although there are many modes of administration, CORT administration in drinking water [154,155,156] or subcutaneous injection [157] are often used in chronic GC administration. Chronic oral administration by drinking water has hardly any impact on animals [158]; however, it is impossible to control the drinking dosage in each animal and this may cause individual differences. The intraperitoneal injection and oral route by gavage are feasible to induce depression in the animal model [159,160]. Furthermore, microinjections in the brain have also been used in rodents, which are beneficial for dosage and stimulation area control [161]. Similarly, as nociceptive stress, they may affect the state of rodents during the experiment. The different doses and cycles of CORT administration can induce different models of neuropsychiatric disorders, such as Gulf War illness [162] and anxiety [122]. On the contrary, GR antagonists can reverse the effect of chronic stress by restoring the functional balance of MR/GR, such as mifepristone [163]. Consequently, the time, dosage, and style of GC administration affect its function.

In conclusion, chronic excess GC administration contributes to HPA axis dysfunction, which further triggers anxiety-like behaviors, depression, and memory loss. This review mainly summarizes behavioral changes and the specific mechanisms of chronic CORT administration-induced animal models in neuropsychiatric disorder research, including neuroinflammatory response, pyroptosis, oxidative stress, neuroplasticity, and apoptosis. Of special interest, chronic CORT administration of different doses and cycles can destroy the balance of the MR/GR ratio to make dose-dependent effects of CORT on the CNS. Hopefully, this review can help researchers to exploit new methods of neuropsychiatric treatment and further understand the pathogenic mechanism of mood disorders.

## Figures and Tables

**Table 1:** Chronic CORT-induced behavioral changes.

Species	Gender	Weight	Age	Intervention	Behavior Tests	Results	References
*C57BL/6 mice*	Male		7–8 weeks old	CORT (40 mg/kg, s.c.) for 36 days	OFT, EMP, FST, TST	Anxiety↑ Depression↑	Wang, G. et al. 2024 [19]
*C57BL/6 mice*	Male and female		5 weeks old	CORT (40 mg/kg, s.c.) for 5 weeks	OFT, EMP, SPT, FST, TST	Depression↑	Tao, Y. et al. 2024 [21]
*C57BL/6 mice*	Male		4 weeks old	CORT (35 μg/mL) in drinking water for 4 weeks	FST, TST	Depression↑	Kim, S. et al. 2024 [22]
*C57BL/6 mice*	Male		6–8 weeks old	CORT (25 μg/mL) in drinking water for 21 days	TST	Depression↑	Ma, H. et al. 2024 [23]
*C57BL/6N mice*	Male		6–8 weeks old	CORT (70 μg/mL equivalent to 5 mg/kg/day) in drinking water for 25 days	OFT, NSF, SST, FST, TST	Depression↑ Memory loss↑	Du, Q. et al. 2024 [24]
*C57BL/6J* *mice*	Male		6–8 weeks old	CORT (40 mg/kg, s.c.) for 5 weeks	SPT, FST, TST	Depression↑	Zhao, M. et al. 2024 [25]
*C57BL/6J* *mice*	Male		8 weeks old	CORT (10 mg/kg, i.p.) between the hours of 09:30 and 11:00 a.m. daily for 21 days	SPT, NSF, SST, FST, TST	Depression↑	Zeng, J. et al. 2024 [26]
*C57BL/6J* *mice*	Male	20–25 g	7–8 weeks old	CORT (70 μg/mL equivalent to 10 mg/kg/day) in drinking water for 21 days	OFT, SPT, FST, TST	Depression↑	Xu, C. et al. 2024 [27]
*C57BL/6J* *mice*	Male		8 weeks old	CORT (20 mg/kg, s.c.) for 21 days	OFT, NSF, SPT, FST, TST	Depression↑	Luo, S. et al. 2024 [28]
*Long Evans rats*	Female	150–250 g		CORT (40 mg/kg, s.c.) between the hours of 08:00 and 11:00 a.m. daily for 21 days	FST	Depression↑	Scheil, K. K. A. et al. 2024 [29]
*Sprague Dawley rats*	Male	350 ± 50 g	6–8 weeks old	CORT (40 mg/kg, s.c.) for 21 days	OFT, NOR, EPM, FST, Groom test	Anxiety↑ Depression↑	Bergosh, M. et al. 2024 [30]
*C57BL/6 mice*	Male	18–22 g		CORT (20 mg/kg, s.c.) every day for 3 weeks	OFT, SPT, TST	Depression↑	Zhang, L. et al. 2023 [31]
*C57BL/6J mice*	Male	18–22 g		CORT (20 mg/kg, i.p.) for 21 consecutive days	FST, TST	Depression↑	He, J. et al. 2023 [32]
*C57BL/6J mice*	Male		7 weeks old	CORT (35 μg/mL equivalent to 5 mg/kg/day) in drinking water for 10 weeks	OFT, NSF, SST	Depression↑	MUSAEL Musaelyan, K. et al. 2023 [33]
*ICR mice*	Male	26 ± 2 g	7 weeks old	CORT (40 mg/kg, s.c.) for 6 weeks	EPM, SPT, NSF, FST, TST	Anxiety↑ Depression↑	Wang, Z. et al. 2023 [34]
*C57BL/6J mice*	Male			CORT (20 mg/kg, s.c.) for 21 consecutive days	SPT, FST, TST	Depression↑	Wang, X. L. et al. 2023 [35]
*C57BL/6N mice*	Male		8–10 weeks old	CORT (20 mg/kg, oral gavage) every day for 28 days	OFT, SPT, FST, TST	Depression↑	Qin, Z. et al. 2023 [36]
*Rats*	Male	190–200 g	7–8 weeks old	CORT (20 mg/kg, i.v.) for 28 days	LDT, EPM, FST, MWM	Anxiety↑ Depression↑ Memory loss↑	Samad, N. et al. 2023 [18]
*Albino Swiss CD-1 mice*	Male	21 ± 2 g	6 weeks old	CORT (20 mg/kg, s.c.) for 21 days	SPT, FST	Depression↑	Głuch-Lutwin, M. et al. 2023 [37]
*C57BL/6J mice*	Female	18.85 ± 0.16 g	6–8 weeks old	CORT (35 μg/mL equivalent to 5 mg/kg/day) in drinking water for 7 weeks	OFT, NSF, FST	Anxiety↑ Depression↑	Ramadan, B. et al. 2022 [38]
*ICR mice*	Male	25–28 g	7 weeks old	CORT (40 mg/kg, i.p.) once daily for 21 consecutive days	OFT, PAT, SPT, FST, TST	Depression↑ Memory loss↑	Lim, D. W. et al. 2022 [39]
*C57BL/6 mice*	Male	24.38 ± 2.05 g	5 weeks old	CORT (40 mg/kg, s.c.) once a day for 14 days	SPT, FST, TST	Depression↑	Su, B. et al. 2022 [40]
*Swiss mice*	Male	23–26 g		CORT (20 mg/kg, s.c.) for 3 consecutive weeks	OFT, EPM, TST, FST	Depression↑	Bai, G. et al. 2022 [41]
*C57BL/6J mice*	Male		6 weeks old	CORT (20 mg/kg, s.c.) for 3 weeks	OFT, SIT, NSF	Anxiety↑ Depression↑	Yang, Y. et al. 2022 [42]
*C57BL/6 mice*	Male	20–22 g	8 weeks old	CORT (20 mg/kg, s.c.) for 3 weeks	OFT, SPT, FST	Depression↑	Chai, Y. et al. 2022 [43]
*C57BL/6 mice*				CORT (40 mg/kg, i.p.) for 3 weeks	OFT, FST, TST, MWM	Depression↑ Memory loss↑	LI H et al. 2022 [20]
*C57BL/6 mice*	Male		7 weeks old	CORT (20 mg/kg, s.c.) for 6 weeks	OFT, SPT, FST	Depression↑	Gao ZY et al. 2022 [16]
*ICR mice*	Male	18–22 g	6–8 weeks old	CORT (40 mg/kg, s.c.) for 3 weeks	SPT, FST	Depression↑	Zhang, C. et al. 2022 [44]
*ICR mice*	Male	30 ± 2 g	7 weeks old	CORT (20 mg/kg, s.c.) for 3 weeks	OFT, SPT, FST, TST	Depression↑	Huang, J. et al. 2022 [45]
*ICR mice*	Male	18–22 g	6–8 weeks old	CORT (20 mg/kg, i.p.) for 4 weeks	OFT, SPT, FST, TST	Anxiety↑ Depression↑	Sun, J. Y. et al. 2022 [46]
*Swiss mice*	Male	25–30 g		CORT (20 mg/kg, s.c.) between 09:00 and 11:30 a.m. for 21 consecutive days	NOR, YMT, SST, SPT, FST, TST,	Depression↑ Memory loss↑	Oliveira, T. Q. et al. 2022 [47]
*Swiss mice*	Male			CORT (20 mg/kg, s.c.) for 21 days	OFT, FST	Depression↑	Shuster, A. L. et al. 2022 [48]
*Swiss mice*	Female	19–23 g	8–10 weeks old	CORT (20 mg/kg, s.c.) for 21 days	OFT, SDA Test, YMT, SPT, FST	Depression↑ Memory loss↑	MAIA Maia Oliveira, I. C. et al. 2022 [49]
*SAMP8 mice*	Male		6 months old	CORT (40 mg/kg, s.c.) for 21 days	OFT, FST	Depression↑	Chou, M. Y. et al. 2022 [50]
*Kunming mice*	Male	18–22 g		CORT (20 mg/kg, s.c.) for 21 days	OFT, EPM, FST, TST	Depression↑	Bai, G. et al. 2022 [51]
*Long Evans rats*	Male			CORT (40 mg/kg, s.c.) for 21 days	OFT, FST	Depression↑	Allen, J. et al. 2022 [52]
*ddY mice*	Male		7 weeks old	CORT (40 mg/kg, s.c.) for 14 days	SLA, BMT, FST	Depression↑	Araki, R. et al. 2021 [53]
*C57BL/6J mice*	Male and female		9–10 weeks old	CORT (20 mg/kg, i.p.) for 10 days	SPT, FST	Depression↑	Patel, S. D. et al. 2021 [54]
*C57BL/6J mice*	Male		5 weeks old	CORT (20 mg/kg, s.c.) between 09:00 and 09:30 a.m. daily for 28 days	SPT, FST, TST	Depression↑	Zhang, D. et al. 2021 [55]
*FVB wild-type mice*	Male	20–25 g	6–8 weeks old	CORT (400 μg/mL) in drinking water for 21 days	SPT, FST	Depression↑	Luo, S. et al. 2021 [56]
*Long Evans rats*	Male	225–250 g		CORT (40 mg/kg, s.c.) for 21 days	NOR, FST, PPI	Depression↑ Memory loss↑	Brymer, K. J. et al. 2021 [57]
*Wistar rats*	Male		6 weeks old	CORT (40 mg/kg, s.c.) for 4 weeks	SPT, FST,	Depression↑	Hao, Y. et al. 2021 [58]
*Swiss mice*	Female	22–25 g	8–10 weeks old	CORT (20 mg/kg, s.c.) for 22 consecutive days	YMT, SDIT, SIT, PPI	Memory loss↑	Chaves, R. C. et al. 2020 [59]
*Swiss mice*	Male	30–40 g	45–60 days old	CORT (20 mg/kg) in drinking water for 21 days	OFT, TST	Depression↑	Camargo, A. et al. 2020 [60]
*Swiss albino mice*	Male	25–30 g		CORT (20 mg/kg, s.c.) for 21 days	NOR, MWM, object in place test	Memory loss↑	K, V. A. et al. 2020 [61]
*APP/PS1 Tg mice, C57BL/6J mice*			5 months old	CORT (10 mg/kg, s.c.) for 3 months	MWM, nest construction	Memory loss↑	Zhang, S. Q. et al. 2020 [62]
*C57BL/6J mice*	Male		5 weeks old	CORT (20 mg/kg, s.c.) for 14 days	SLA, FST, female encounter test	Depression↑	YOKO Yokoyama, R. et al. 2020 [63]
*C57BL/6J mice*	Male			CORT (20 mg/kg, s.c.) for 6 weeks	OFT, FST, TST	Depression↑	Xie, X. et al. 2020 [64]
*C57BL/6J mice*	Female	18–22 g	6–8 weeks old	CORT (40 mg/kg, s.c.) for 6 weeks	OFT, SPT, FST, TST, NSF	Depression↑	Zhao, F. et al. 2020 [65]
*C57BL/6 mice*	Male		6 weeks old	CORT (20 mg/kg, s.c.) for 6 weeks	FST, TST	Depression↑	Xie, X. et al. 2020 [66]
*Heterozygous Reeler mice and wild-type mice*	Male and female		6 weeks old	CORT (25 mg/L) in drinking water for 3 weeks	YMT, FST, PPI and startle reactivity, drug-induced hyper-locomotor activity	Depression↑ Memory loss↑	Notaras, M. J.et al. 2020 [67]
*Sprague Dawley rats*	Male	220–250 g		CORT (40 mg/kg, s.c.) for 21 days	SPT, FST, MWM	Depression↑ Memory loss↑	Zhao, Y. et al. 2020 [68]
*Sprague Dawley rats*	Male	180–220 g		CORT (20 mg/kg, s.c.) for 6 weeks	OFT, SPT, MWM	Anxiety↑ Depression↑ Memory loss↑	Li, Z. et al. 2020 [69]
*Long Evans rats*	Male	225–250 g	7 weeks old	repeated and cyclic CORT (20 mg/kg, s.c.) between 9 and 11 a.m. for 21 days	OFT, SPT, FST	Depression↑	Lebedeva, K. A. et al. 2020 [70]
*Wistar rats*	Male	180 ± 20 g		CORT (40 mg/kg, s.c.) for 21 days	OFT, SPT, FST	Depression↑	Yu, Z. et al. 2020 [71]
*Swiss mice*	Female	21–25 g	8–10 weeks old	CORT (20 mg/kg, s.c.) for 23 days	OFT, LDT, HBT, EPM, YMT, SIT, SPT, FST, TST	Anxiety↑ Depression↑ Memory loss↑	Capibaribe, V. C. C. et al. 2019 [72]
*C57BL/6J mice*	Male	18–22 g		CORT (40 mg/kg, s.c.) for 21 days	OFT, NOR, MWM, TST	Depression↑ Memory loss↑	Chen, H. et al. 2019 [73]
*C57BL/6 mice*	Male	18–22 g	8–10 weeks old	CORT (40 mg/kg, s.c.) between 8:00 and 10:00 a.m. for 8 weeks	EPM, FST, TST	Anxiety↑ Depression↑	Zhang, K. et al. 2019 [74]
*Swiss mice*	Female	22–25 g	8–10 weeks old	CORT (20 mg/kg, s.c.) daily for 21 days	OFT, EPM, SPT, FST, TST	Anxiety↑ Depression↑	Chaves, R. C. et al. 2019 [75]
*Wistar rats*	Male	~200 g	6 weeks old	CORT (40 mg/kg, s.c.) for 21 consecutive days	OFT, SPT, FST	Depression↑	Shen, Q. et al. 2019 [76]
*C57BL/6 mice*	Male		5 weeks old	CORT (100 μg /mL) in drinking water for 14 days, CORT (50 and 25 μg /mL) in drinking water for 3 days	OFT, YMT, NOR, SPT, FST, TST	Depression↑ Memory loss↑	Murata, K. et al. 2018 [77]
*Swiss mice*	Female	22–25 g		CORT (20 mg/kg, s.c.) between 9:00 and 10:30 a.m. for consecutive 21 days	OFT, YMT, EPM, SPT, FST, TST	Anxiety↑ Depression↑ Memory loss↑	Lopes, I. S. et al. 2018 [78]
*Swiss mice*	Female	30–32 g		CORT (20 mg/kg, s.c.) between 9:00 and 11:30 a.m. for 21 days	YMT, NOR, SIT, TST	Depression↑ Memory loss↑	de Sousa, C. N. S. et al. 2018 [79]
*Swiss mice*	Male	30–40 g		CORT (20 mg/kg, orally administration) for 21 days	OFT, SST, FST, TST	Depression↑	Camargo, A.et al. 2018 [80]
*Swiss albino mice*	Male	26–32 g		CORT (20 mg/kg, s.c.) for 21 days	OFT, EPM, SPT, SST, NSF, FST, TST	Anxiety↑ Depression↑	Kv, A. et al. 2018 [81]
*ICR mice*	Male	18–22 g		CORT (20 mg/kg, s.c.) for 21 days	OFT, SPT, FST, TST	Depression↑	Bai, Y. et al. 2018 [82]
*CD Sprague Dawley rats*	Female		3 months old	CORT (40 mg/kg, s.c.) between 9:30 and 11:30 a.m. for 21 days	OFT, FST, maternal care	Depression↑	Kott, J. M. et al. 2018 [83]
*C57BL/6 mice*	Male	24 ± 2 g	5 weeks old	CORT (40 mg/kg, s.c.) between 9:00 and 11:00 a.m. for 21 consecutive days	SPT, TST	Depression↑	Wang, S. S. et al. 2017 [84]
*C57BL/6NTac mice*	Male	25–30 g	7–8 weeks old	CORT (35 μg/mL equivalent to 5 mg/kg/day) in drinking water for 8 weeks	OFT, EPM, SPT, NSF, FST, TST	Anxiety↑ Depression↑	Mendez-David, I. et al. 2017 [85]
*Swiss mice*	Male	25–30 g		CORT (20 mg/kg, s.c.) between 9:00 and 11:30 a.m. for 21 days	OFT, EPM, SPT, TST, sleeping time test, rota rod test	Anxiety↑ Depression↑	Oliveira, T. Q. et al. 2017 [86]
*Swiss mice*	Female	30–40 g	40–45 days old	CORT (20 mg/kg, oral gavage) for 21 days	OFT, FST, TST	Depression↑	Pazini, F. L. et al. 2017 [87]
*Long Evans rats*	Male	200–250 g		CORT (20 and 40 mg/kg, s.c.) for 21 consecutive days	FST	Depression↑	Lebedeva, K. A. et al. 2017 [88]
*Sprague Dawley rats*	Male	Adolescent (90 ± 5 g), adult (350 ± 20 g)	Adolescent (28–29 days), adult (70–71 days)	CORT (40 mg/kg, i.p.) between 9:00 and 11:00 a.m. for 21 days	OFT, MWM, SLA, SPT, PPI	Anxiety↑ Depression↑ Memory loss↑	Li, J. et al. 2017 [89]
*C57BL/6NTac mice*	Male		8 weeks old	CORT (10 mg/kg) for 3 weeks	EPM, NSF, SST, FST	Anxiety↑ Depression↑	Brachman, R. A. et al. 2016 [90]
*C57BL/6NTac mice*	Male	20–25 g	8 weeks old	CORT (35 μg/mL equivalent to 5 mg/kg/day) in drinking water for 4 weeks	OFT, EPM, NSF, SST	Anxiety↑ Depression↑	Siopi, E. et al. 2016 [91]
*C57BL/6JRj mice*	Male		8–10 weeks old	CORT (35 μg/mL equivalent to 5 mg/kg/day) in drinking water for 4 weeks	OFT, NOR, FC, BMT, SST	Anxiety↑ Depression↑ Memory loss↑	Darcet, F. et al. 2016 [92]
*BALB/c mice*	Female			CORT (35 μg/mL) in drinking water for 21 days	SPT, FST	Depression↑	Nashed, M. G. et al. 2016 [93]
*Wistar rats*	Male		8 weeks old	CORT (200 μg/mL equivalent to ∼27 ± 1.6 mg/kg/day) in drinking water for 21 days	OFT, EPM, predator odor test	Anxiety↑	Govic, A. et al. 2016 [94]
*Sprague Dawley rats*	Female		77–84 days old	CORT (200 mg, pellet implantation); CORT (40 mg/kg, s.c.) between 8:00 and 10:00 a.m. for 23 days; CORT (200 μg /mL) in drinking water for 20 days, CORT (150 μg /mL) in drinking water for 1 day, CORT (100 μg /mL) in drinking water for 1 day, CORT (50 μg /mL) in drinking water for 1 day	OFT, FST	Depression↑	Kott, J. M. et al. 2016 [95]
*C57BL/6J mice*		25–35 g	7 weeks old	CORT (35 μg/mL equivalent to 5 mg/kg/day) in drinking water for 21 days	OFT, NOR, FC, object location test, EPM, NSF, SST, SPT, TST,	Anxiety↑ Depression↑ Memory loss↑	Quesseveur, G. et al. 2015 [96]
*C57BL/6NTac mice*	Male	23–25 g	7–8 weeks old	CORT (35 mg/mL) in drinking water for 4 weeks	OFT, EPM, NSF, SST	Anxiety↑ Depression↑	Mendez-David, I. et al. 2015 [97]
*BALB/c mice*	Female		4–6 weeks old	CORT (35 μg/mL equivalent to 6.53 ± 0.16 mg/kg/day) in drinking water for 21 days	SPT, FST, TST	Depression↑	Nashed, M. G. et al. 2015 [98]
*iBax mice*	Male		8–10 weeks old	CORT (70 μg/mL equivalent to 10 mg/kg/day) in drinking water for 21 days	OFT, EPM, NSF, FST, TST	Anxiety↑ Depression↑	Hill, A. S. et al. 2015 [99]
*Wild-type mice*	Male		8 weeks old	CORT (35 μg/mL) in drinking water for 6 weeks	NSF, splash grooming test	Anxiety↑ Depression↑	Schloesser, R. J. et al. 2015 [100]
*HRM and wild-type* *mice*	Male and female		6 weeks old	CORT (50 mg/L) in drinking water for 21 days	YMT, NOR, SIT, PPI	Memory loss↑	Schroeder, A. et al. 2015 [101]
*Swiss mice*	Female	30–32 g		CORT (20 mg/kg, s.c.) between 9:00 and 11:30 a.m. for 21 days	SPT, FST	Depression↑	de Sousa, C. N. et al. 2015 [102]
*Swiss albino mice*	Male	25–30 g	4–6 weeks old	CORT (40 mg/kg, s.c.) for 21 days	SPT, FST, TST	Depression↑	Ali, S. H. et al. 2015 [103]
*Swiss albino mice*	Male	20–25 g	11–12 weeks old	CORT (40 mg/kg, s.c.) for 28 days	SLA, LDT, FST	Depression↑	Gupta, D. et al. 2015 [104]
*Fischer 344 rats*	Male	250–320 g		CORT (30 μg, stereotaxic implantation)	EPM	Anxiety↑	Tran, L. et al. 2015 [105]
*Sprague Dawley rats*	Male		3–4 weeks old	CORT (50 μg/mL equivalent to 7.1 mg/kg/day) in drinking water for 21 days	NSF, SPT	Depression↑	Kvarta, M. D. et al. 2015 [106]
*Sprague Dawley rats*	Male	325–350 g		CORT (50 mg/kg, s.c.) for 21 days	OFT, SPT, FST	Depression↑	Dwivedi, Y. et al. 2015 [107]

BMT—Barnes maze test; EPM—elevated plus maze; FC—fear conditioning; FST—forced swim test; HBT—hole-board test; LDT—light/dark test; NOR—novel object recognition test; NSF—novelty-suppressed feeding test; OFT—open field test; PAT—passive avoidance test; PPI—prepulse inhibition test; SDIT—step-down avoidance test; SIT—social interaction test; SLA—spontaneous locomotor activity; SPT—sucrose preference test; SST—sucrose splash test; TST—tail suspension test; YMT—Y-maze test; s.c.—subcutaneous injection; i.p.—intraperitoneal injection; i.v.—intravenous injection; ↑—Exhibiting corresponding behaviors.

## Abbreviations

ACTH	Adrenocorticotropic hormone
AMPA	α-amino-3-hydroxyl-5-methyl-4-isoxazole-propionate
ASC	Apoptosis-associated speck-like protein containing CARD
ATF4	Activating transcription factor 4
BDNF	Brain-derived neurotrophic factor
BLA	Basolateral amygdala
BMT	Barnes maze test
Capn2	Calpain 2
Cnp	C-type natriuretic peptide
CNS	Central nervous system
CORT	Corticosterone
Cox	Cytochrome oxidase
CPP	Conditioned place preference
DG	Dentate gyrus
eIF2α	Eukaryotic translation initiation factor-2α
EPM	Elevated plus maze
ER	Endoplasmic reticulum
EZM	Elevated zero maze
FC	Fear conditioning
FKBP5	FK506-binding protein 5
FST	Forced swim test
GC	Glucocorticoid
GDH	Glutamate dehydrogenase
GR	Glucocorticoid receptor
GRE	GC response elements
GSDMD	Pyroptosis executor gasdermin D
HBT	Hole-board test
HPA	Hypothalamus–pituitary–adrenal
HPC	Hippocampus
IDH2	Isocitrate dehydrogenase 2
IL-1	Interleukin-1
IL-6	Interleukin-6
IL-10	Interleukin-10
IL-18	Interleukin-18
i.p.	Intraperitoneal injection
i.v.	Intravenous injection
LDT	Light/dark test
MDA	Malondialdehyde
MERTK	Mer tyrosine kinase
MOMP	Mitochondrial outer membrane permeabilization
mPFC	Medial prefrontal cortex
MR	Mineralocorticoid receptor
ND	NADH-ubiquinone oxidoreductase
NF-κB	Nuclear factor-κB
NLRP3	NLR family, pyrin domain containing 3
NOR	Novel object recognition test
NRF2	Nuclear factor erythroid 2-related factor 2
NSF	Novelty-suppressed feeding test
OFT	Open field test
OS	Oxidative stress
PAT	Passive avoidance test
PERK	Protein kinase RNA-like ER kinase
PPI	Prepulse inhibition test
PSD-95	Postsynaptic density protein 95
PVN	Paraventricular nucleus
ROS	Reactive oxygen species
s.c.	Subcutaneous injection
SOD	Superoxide dismutase
SDIT	Step-down avoidance test
SGK1	Serum- and GC-inducible kinase 1
SIRT	Sirtuin
SIT	Social interaction test
SLA	Spontaneous locomotor activity
SPT	Sucrose preference test
SST	Sucrose splash test
TNF-α	Tumor necrosis factor-α
TST	Tail suspension test
Vamp7	Vesicle-associated membrane protein 7
YMT	Y-maze test
